# Phage-based biocontrol of *Salmonella* and *E. coli* in raw chicken filets: optimizing phage-based solutions to enhance food safety under cooled storing conditions

**DOI:** 10.3389/fmicb.2025.1696826

**Published:** 2026-01-07

**Authors:** Oleksandra Berhilevych, Elisa Peh, Johanna Charlotte Vahle, Madeleine Plötz, Sophie Kittler

**Affiliations:** Institute of Food Quality and Food Safety, University of Veterinary Medicine Hannover, Hannover, Germany

**Keywords:** phage biocontrol, phage cocktail, Appelmans protocol, cold storage, decontamination, antibacterial efficacy

## Abstract

Food safety continues to be an important issue for consumer protection and public health globally. Chicken meat is considered a primary source of *Salmonella* and *E. coli* infections in humans. In recent years, phage-based biocontrol has attracted attention as a promising approach to combat these foodborne pathogens due to its advantages over traditional methods and its biological properties as a natural bactericide. Using phage-based control as a decontamination method to ensure microbial safety of food aligns with the One Health strategy for sustainable pathogen control and prevention of foodborne infections. This study aimed to develop and evaluate the effectiveness of a three-phage cocktail with optimized efficacy for simultaneously controlling *Salmonella* and *E. coli* on raw chicken filets during cold storage. To optimize the efficacy of the final phage cocktail, three phages were selected according to their host ranges and the efficiency of plating (EOP) values. They were combined in a cocktail, and the host range was expanded using the Appelmans protocol for 30 training cycles. The antibacterial efficacy of the trained three-phage cocktail was evaluated in liquid culture using a planktonic killing assay (PKA) and on raw chicken filets stored at 4 ± 0.5 °C for 72 h, employing multiplicities of infection (MOI) of 1, 10, and 100 for targeting filets contaminated with single *Salmonella* and *E. coli* strains and a mixture of both. After training according to the Appelmans protocol, the cocktail showed an expanded host range, covering 62.5% (5/8 after 30 training cycles) instead of 37.5% (3/8 before training) of the tested bacteria. The planktonic killing assay demonstrated that the trained three-phage cocktail had a significant inhibitory effect on bacterial growth of the *Salmonella* strains (4/4, 100%) from 3 to 6 h, while the non-trained initial three-phage cocktail’s effect was less pronounced (1/4, 25%) and lasted only 3 h. However, three of four *E. coli* strains (75%) were not sensitive to the three-phage cocktail after 30 cycles of the Appelmans protocol compared to two out of four strains (50%) with the non-trained initial three-phage cocktail. On raw chicken filets, significant bacterial reduction was observed when using MOI 10 and 100 of the trained three-phage cocktail. A maximum reduction of 1.56 log_10_ CFU/mL of *Salmonella* BfR 20-SA00418 and 1.48 log_10_ CFU/mL of *E. coli* 19/302/1/A after 72 h compared to placebo-treated controls were achieved using an MOI of 100. We observed a synergistic effect of the three-phage cocktail compared to single treatment, with a stronger effect on *Salmonella* than on *E. coli* strains. Using the Appelmans protocol improved the effects of the developed three-phage cocktail, leading to broader pathogen coverage. The efficacy of the developed three-phage cocktail under cold storage conditions and its ability to reduce the bacterial load in raw chicken filets highlighted its potential for extending shelf life and reducing risks for the consumer. The findings of this study demonstrate that the developed and optimized three-phage cocktail is a promising biocontrol agent for enhancing safety in raw chicken meat production.

## 1 Introduction

Globally, health authorities continue to face different challenges in controlling the prevalence of foodborne disease. *Salmonella* and *E. coli* are among the most important foodborne pathogens globally. Appropriate control measures for foodborne infections are urgently needed, and novel ‘natural’ antimicrobial approaches aligned with the One Health strategy. The One Health approach aims to enhance consumer protection and improve public health, interconnecting human, animal, and environmental health. Using the natural enemies of bacteria bacteriophages (phages)—to combat foodborne zoonotic pathogens aligns with improved food safety and the One Health concept (Álvarez and Biosca, 2025).

In recent years, the application of phages has gained attention for use in food safety, particularly in combating major foodborne pathogens. The application across a wide range of food matrices is possible, due to their strict host specificity, bactericidal activity, ability to self-replicate, cost-effectiveness, and lack of effects on product properties. Thus, phages have been evaluated as promising tools on different stages of the food production chain (in preharvest production, food processing, storage, and retail; Alomari et al., 2021; Gambino and Brøndsted, 2021; Hyla et al., 2022).

Several studies suggest that phage application may be effective for reducing *Salmonella* (Kim et al., 2020; Sukumaran et al., 2015; Kumar et al., 2017) and *E. coli* (Minh et al., 2016; Seo et al., 2016) on raw meat and poultry. However, the use of single phages has some limitations: most phages have a narrow host spectrum, and increased development of phage resistance has been observed. To reduce these issues, polyvalent broad-spectrum phages and/or phage cocktails can be used instead of single phages (Haines et al., 2021; Hyla et al., 2022; Poojari et al., 2022; Zhou et al., 2022). Several commercial phage products, such as SalmoFresh™, Salmonelex™, EcoShield™, Finalyse™, STECleanz™, and Armament™ have already been officially approved by the US Food and Drug Administration (FDA) and the US Department of Agriculture (USDA). Most of these commercially produced phages are used in the preharvest control of foodborne pathogens (Alomari et al., 2021; Bumunang et al., 2023).

However, limited studies have reported on the application of phage cocktails for the biocontrol of mixed *Salmonella* and *E. coli* contamination in poultry meat (Poojari et al., 2022) or on the use of polyvalent broad-spectrum phages capable of simultaneously controlling *Salmonella* and *E. coli* in foods (Zhou et al., 2022).

Phage training is a new approach for the development and production of efficient phages. It aims to improve their spectrum of bacterial strains covered, their effectiveness, and/or their stability. Studies show that a well-designed evolutionary training method for phages is promising for obtaining highly efficient phages in practice (Borin et al., 2021; Zhang et al., 2021; Rohde et al., 2018). However, phage training involves the analysis of characterization data for the selection of suitable phages. Using selected phages, various methods for phage training have been developed under laboratory conditions (Vu et al., 2024).

The Appelmans protocol is one of these methods, formerly used by Eastern European researchers to generate therapeutic phages with adapted host ranges. It was developed in the 1920s by Appelmans (Chanishvili and Sharp, 2009). This protocol has been used in various forms for almost a century. It was designed in the Republic of Georgia and was described in detail by Burrowes et al. in 2019. It is suitable to adapt phage cocktails to a set of bacterial strains. The application of several phages to a diverse set of host strains with different susceptibility degrees can foster spontaneous mutations and recombinations that can influence the host range of the phages. This technique was effectively used for phages against *P. aeruginosa* clinical isolates (multi-drug-resistant isolates that produced high amounts of biofilm), carbapenem-resistant *Acinetobacter baumannii* (CRAB), and *Staphylococcus aureus* (Vu et al., 2024).

The work of this study followed up on knowledge gained in previous studies where the effectiveness of phages was investigated both *in vitro* and in challenge tests (Kittler et al., 2020; Korf et al., 2020; Shakeri et al., 2021). In contrast to previous studies, our approach focuses on developing a three-phage cocktail with optimized simultaneous polyvalent efficacy against *Salmonella* and *E. coli*.

## 2 Materials and methods

### 2.1 Bacterial strains

A total of 23 bacterial strains were examined in this study, including 13 *Salmonella* and 10 *E. coli* strains (six of which were ESBL strains). Most of the bacterial strains were isolated from food products and originate from the collection of the Institute of Food Quality and Food Safety, University of Veterinary Medicine Hannover, Germany (Supplementary Table S1), while E28 was a strain derived from a previous project (Korf et al., 2020). Before starting the experiments, the bacterial strains were streaked onto Columbia agar supplemented with sheep blood (Oxoid Deutschland GmbH, Wesel, Germany) and incubated aerobically at 37 ± 0.5 °C overnight.

### 2.2 Phages, host range analysis, and the efficiency of plating determination

Sixteen phages (15 phages against *E. coli* and one phage against *Salmonella*) from the collection of the Institute of Food Quality and Food Safety were included in the experiments to identify suitable candidates for a phage cocktail (Supplementary Table S2). The host ranges of the phages were assessed according to the overlay method described by Korf et al. (2020), with some modifications. Briefly, plates with 1.5% LB-base agar (Carl Roth GmbH+Co. KG, Karlsruhe, Germany) were overlaid with 5 mL of 0.7% LB—soft agar, containing 100 μL of the respective phage suspension (1 × 10^8^–1 × 10^9^ PFU/mL; *n* = 17) and 100 μL of a McFarland standard three-culture suspension of the respective bacterial strain (1 × 10^9^ CFU/mL, *n* = 23). After overnight aerobic incubation at 37 ± 0.5 °C, the presence of plaques was assessed. All combinations of phages and bacterial strains were tested in triplicate. Phages that produced plaques were used to calculate the EOP by dividing the concentration on the tested bacterial strains by the concentration measured on the host strains of the respective phage. The relative EOP was graded according to the number of plaques visible on the agar plates: high sensitivity EOP ≥ 0.5; moderate sensitivity 0.1 ≤ EOP ≤ 0.5; and low sensitivity 0.001 ≤ EOP ≤ 0.1 (Khan Mirzaei and Nilsson, 2015).

### 2.3 Expanding the host range using the Appelmans protocol

To expand the host range of the phage cocktail, three phages and eight bacterial strains were co-incubated according to the Appelmans protocol described by Burrowes et al. (2019). Briefly, for the first round of the method, three phages were combined in a 1:1:1 ratio at a total concentration of 1 × 10^9^ PFU/mL. The concentrations of all three phages were adjusted before mixing to ensure similar titers for each phage (approximately 3.3 × 10⁸ PFU/mL). Serial 10-fold dilutions of the cocktail were prepared in LB broth (Carl Roth GmbH+Co. KG, Karlsruhe, Germany). Subsequently, 100 μL of the serial dilution of the cocktail in LB broth was added to the wells of a 96-well microtiter plate (Sarstedt, Germany). Subsequently, 100 μL of LB broth was mixed with 10 μL of the overnight culture, to achieve a density equivalent to McFarland standard 3. Column 1 in the layout was used as bacterial control (bacteria only), column 2 was left empty, and column 3 served as a negative control (LB broth only). The microtiter plate was incubated overnight at 37 ± 0.5 °C on a shaking platform at 200 rpm. After incubation, the turbidity of the wells was assessed. The first well showing complete lysis (clear broth medium) and the next dilution with incomplete lysis were pooled. If no lysis was observed, wells with the highest phage concentration were pooled. The pooled lysates were centrifuged at 4500 × g for 15 min. The solution was filtered using a 0.45 μm syringe filter (Carl Roth GmbH+Co. KG, Karlsruhe, Germany) and stored at 4 ± 0.5 °C until it was used for the next round of co-incubation, using the same experimental design. The host range of the pooled lysate and the phage concentrations at 0, 3, 6, 12, 20, and 30 cycles of the Appelmans protocol were measured using the overlay method.

### 2.4 Efficacy of expanded host range three-phage cocktail in liquid culture (planktonic killing assay, PKA)

After 30 rounds of phage training using the Appelmans protocol, the efficacy of the three-phage cocktail was examined with four *Salmonella* strains, including one original host, and 6 *E. coli* strains, including two original hosts, using a planktonic killing assay (PKA). Experiments were carried out using the Tecan Spark automatic microplate reader (Tecan Austria GmbH, Grödig, Austria) via optical density (OD) absorbance measurements at 600 nm (OD_600_), using flat-bottom 96-well plates (Sarstedt, Germany). Tests were performed as described by Peh et al. (2023) with minor modifications. Briefly, 100 μL of overnight bacterial cultures (1 × 10^6^ CFU/mL) were added to the wells of a 96-microtiter plate, then 100 μL of phage cocktail suspension (1 × 10^8^ PFU/mL) was added. Growth inhibition of the tested strains was investigated via OD absorbance measurements at 600 nm (OD_600_) using a Tecan Spark automatic microplate reader with an integrated Gas Control Module (Tecan Austria GmbH, Grödig, Austria) at 37 ± 0.5 °C. OD_600_ was measured automatically every 60 min for 24 h. Two controls were included: (i) a growth control without phages and (ii) a negative control (LB broth only). There were two replication wells per microtiter plate, and the experiment was performed in triplicate. The average values of the three repetitions were used for calculations. The area under the curve (AUC) values were determined by spline fitting the control and treatment curves from 0 to 24 h.

### 2.5 Efficacy of expanded host range three-phage cocktail in raw chicken filets

#### 2.5.1 Preparation of meat samples, bacteria, and three-phage cocktail for application in challenge experiments

The antibacterial efficacy of the three-phage cocktail against *Salmonella* and *E. coli* in raw chicken filets was tested during storage at 4 ± 0.5 °C for 72 h. The *Salmonella* strain BfR 20-SA00418 and the *E. coli* strain 19/302/1/A were used for spiking chicken breast filets. Raw meat samples were purchased from a local retail store (Hannover, Germany). The samples were cut into small pieces measuring 6.25 cm^2^ (2.5 cm × 2.5 cm) and placed into the Petri dishes. Before the experiments, all chicken filet samples were investigated for the presence of phages effective against *Salmonella* BfR 20-SA00418 and *E. coli* 19/302/1/A. For that, 2.0 mL of a NaCl-pepton rinse sample from each meat sample was filtered using syringe filters. Of this filtrate, 100 μL were plated on LB agar together with 100 μL of the tested bacterial culture to monitor the presence of phages using the soft-agar overlay method.

Bacterial cultures for spiking were prepared from overnight cultures on Columbia agar supplemented with sheep blood. Bacterial concentrations were adjusted to an OD_600_ of 0.1, corresponding to 1 × 10^8^–1.5 × 10^8^ CFU/mL. Bacterial concentrations were confirmed by plating and counting serial dilutions on LB agar before the experiments. Subsequently, the bacterial suspension was diluted in 0.9% NaCl to obtain a concentration of 1 × 10^4^ CFU/mL.

The three-phage cocktail was diluted in SM buffer to obtain total concentrations of 1 × 10^4^ CFU/mL, 1 × 10^5^ CFU/mL, and 1 × 10^6^ CFU/mL to study the phage’s efficacy at MOIs of 1, 10, and 100.

#### 2.5.2 Study design on raw meat samples

The prepared meat samples were divided into four experimental groups and a control group.

Experimental group A was used to evaluate the antibacterial efficacy of the three-phage cocktail after 30 rounds of the Appelmans protocol against the *Salmonella* BfR 20-SA00418. For this, 100 μL of the bacterial suspension of the *Salmonella* strain in LB broth (1 × 10^4^ CFU/mL) was dropped on each piece of chicken meat of this group.

Experimental group B was used to assess the cocktail’s efficacy against *E. coli* 19/302/1/A. For this, 100 μL of the *E. coli* suspension in LB broth (1 × 10^4^ CFU/mL) was added to each piece of chicken meat in this group.

Experimental groups C and D were designed to test the phage cocktail against a mixture of both bacterial strains. For this, 100 μL of the bacterial suspension of *Salmonella* BfR 20-SA00418 and 100 μL of the suspension of *E. coli* 19/302/1/A in LB broth with a concentration of 1 × 10^4^ CFU/mL of each bacterium were dropped on each piece of chicken meat of this group.

Then, 100 μL of the three-phage cocktail at a multiplicity of infection (MOI) of 1, 10, and 100 were applied on each chicken breast filet of the experimental groups.

In the control groups, 100 μL of SM buffer was added. After the bacteria and the three-phage cocktail application, the chicken meat was stored at 4 ± 0.5 °C for 72 h in the fridge.

#### 2.5.3 Monitoring of bacterial reductions and phage titers on raw chicken filets during cold storage

Pieces of chicken meat from each group were examined after 0, 24, 48, and 72 h of storage. The meat samples were shaken by vortexing with 2.0 mL NaCl-pepton. The rinses were investigated, and the tested bacteria were enumerated on XLD agar (for *Salmonella* BfR 20-SA00418), on TBX agar (for *E. coli*), and on TBX agar with tellurite and neomycin (for *E. coli* 19/302/1/A) by serial dilutions in NaCl-pepton and then counted after overnight incubation at 37 ± 0.5 °C. All agar plates used were produced by Carl Roth GmbH+Co: KG, Karlsruhe, Germany. After incubation in an aerobic atmosphere at 37 ± 0.5 °C for 24 h, grown colonies were enumerated. The colonies were enumerated and reported as log_10_ CFU/mL.

To determine the phage concentrations, the rinses were centrifuged at 4000 × g for 10 min, and the supernatant was filtered through a 0.45 μm membrane. The filtrate was 10-fold diluted, and 100 μL of the appropriate dilution, as well as 100 μL of bacterial host cultures (10^9^ CFU/mL), were transferred to 5 mL of molten LB overlay agar, vortexed, and poured onto the LB base agar plates. The plates were incubated for 24 h. The number of plaques was determined and reported as log_10_ PFU/mL.

### 2.6 Statistical analysis

The data are presented as the mean ± standard error of the mean (SEM) of three independent experiments. The microbial data generated were log_10_-transformed and analyzed using a non-parametric test. Statistical analysis was performed using GraphPad Prism software version 10.4.2 (San Diego, California, United States). Comparisons between several groups (evaluation of the antimicrobial efficacy of a phage cocktail on raw meat) were carried out using the Kruskal-Wallis test, followed by Dunn’s multiple comparison test for values with the nonparametric distribution. In all tests, *p* < 0.05 (*), *p* < 0.01 (**), or *p* < 0.001 (***) were considered statistically significant.

## 3 Results

### 3.1 Host range analysis and EOP determination

The host ranges of 16 phages were examined on *Salmonella* (13 strains) and *E. coli* (10 strains). The host ranges of the tested phages were variable, covering 4.35 to 26.09% of the tested strains. The majority of phages, 12/17 (70.6%), showed plaque formation on *E. coli* strains, whereas only a smaller proportion, 4/17 (23.5%), showed plaque formation on *Salmonella* strains. Comprehensive results on host range and EOP are presented in Supplementary Table S3. The results indicate that among the tested phages, three phages exhibited a broad host range to both *E. coli* and *Salmonella* (3/17, 17.65%), affecting five bacterial strains overall. The EOP analyses showed that most of the tested phages had rather low or moderate EOPs against the tested bacterial strains, and only the phages vB_Eco_LmqsKl 33–12 and vB_Eco_LmqsKl 33–19 showed higher EOP values.

Of the 17 phages, three were selected for further investigation. These phages were chosen for further investigation based on their host ranges, as shown in Table 1. The phage vB_Eco_LmqsKl31-21 exhibited lytic activity against multiple *E. coli* strains (6/10, 60.0%), including ESBL strains (3/6, 50.0%), with a moderate to low effectiveness. The phage vB_Eco_LmqsKl33-12 displayed activity against both *E. coli* (3/10, 30.0%) and *Salmonella* (1/13, 7.7%) strains with moderate and high EOP values, respectively. The phage vB_Sty-LmqsSP6 demonstrated specific moderate sensitivity to a single *Salmonella* strain (1/13, 7.7%).

**Table 1:** Host range characteristics of selected phages determination.

Phage strains	Bacterial strains	Host range analysis			
		Bacterial fraction covered	Efficiency of plating (EOP)*		
			EOP ≥ 0.5	0.1 ≤ EOP ≤ 0.5	0.001 ≤ EOP ≤ 0.1
vB_Eco_LmqsKl31-21	*E. coli* strains	6/10, (60.0%)	-	3/10, (3.0%)	3/10, (3.0%)
	ESBL	3/6, (50.0%)	-	2/6, (33.3%)	1/6, (16.7%)
vB_Eco_LmqsKl33-12	*Salmonella* strains	1/13, (7.7%)	1/13, (7.7%)	-	-
	*E. coli* strains	3/10, (30.0%)		3/10, (30.0%)	-
	ESBL	1/6, (16.7%)		1/6, (16.7%)	-
vB_Sty-LmqsSP6	*Salmonella* strains	1/13, (7.7%)	-	1/13, (7.7%)	-

*Phages with EOP ≥ 0.5 were considered as phages with high sensitivity, phages with 0.1 ≤ EOP ≤ 0.5 as phages with moderate sensitivity, and phages with 0.001 ≤ EOP ≤ 0.1 as phages with low sensitivity.

### 3.2 Host range expansion of the three-phage cocktail

To expand the host range of the selected three phages, the Appelmans protocol was used, exposing eight bacterial strains to a 1:1:1 cocktail of these three selected phages separately (see Tables 2, 3 for strain information).

**Table 2:** Phage stocks used for training according to the Appelmans protocol and their characteristics.

Phage	Titer, PFU/mL	Original host	Host range
vB_Eco_LmqsKl31-21	8,5 × 10^9^	*E. coli* 3981/1/22	*E. coli*, including ESBL strains
vB_Eco_LmqsK33-12	3,62 × 10^9^	*E. coli* 3617/1/22	different serotypes of *Salmonella* and *E. coli*, including ESBL strains
vB_Sty-LmqsSP6	4,41 × 10^9^	*Salmonella* LT2	different serotypes of *Salmonella*

**Table 3:** Bacterial strains used for training according to the Appelmans protocol and their characteristics.

Bacterial strains	Serotype and resistance	Susceptibility to phages (Table 2) before Appelmans protocol
*Salmonella* BfR 19-SA02184	*S. Indiana*	non-susceptible
*Salmonella* BfR 20-SA01020	*S. typhimurium*	non-susceptible
*Salmonella* BfR 20-SA02231	*S. enteritidis*	non-susceptible
*Salmonella* BfR 20-SA00418	*S. enterica* subsp*. enterica* rough variant	susceptible to phage vB_Eco_LmqsK33-12 and phage vB_Sty-LmqsSP6
*E. coli* 19/302/1/A	O2	susceptible to phage vB_Eco_LmqsK33-12
ESBL *E. coli* 716	Cefotaxim resistance (MIC 19 μg/mL)	susceptible to phage vB_Eco_LmqsK31-21
ESBL *E. coli* 290.1	Ceftazidim and Cefotaxim resistance (both MIC 4 μg/mL)	non-susceptible
ESBL *E. coli* 271	Ceftazidim and Cefotaxim resistance (both MIC 8 μg/mL)	non-susceptible

Before using the Appelmans protocol, three out of the eight bacterial strains (37.5%) were sensitive to the individual phages in the cocktail. After serial exposure and pooling of the phage cocktail to the selected bacterial strains in the Appelmans protocol, the host range of the cocktail increased, now covering 62.5% (5/8) of the tested bacteria (Table 4). Specifically, the expanded three-phage cocktail was still unable to lyse two out of the eight strains (25%), and ESBL *E.coli* 716, a previously susceptible strain, lost susceptibility to the cocktail. The host range of the expanded three-phage cocktail was tested using the three original (isolation) hosts. After performing three cycles of the Appelmans protocol, two of the original host bacteria, *E. coli* 3981/1/22 and *E. coli* 3617/1/22, were no longer affected by the evolved three-phage cocktail.

**Table 4:** Susceptibility of bacteria used in the Appelmans protocol and after 30 cycles of phage training.

Susceptibility of bacteria used	Before training				After 30 training cycles	
	Susceptible to			not susceptible to any cocktail phage	Susceptible to phage cocktail	not susceptible to cocktails
	vB_Eco_LmqsKl 31–21	vB_Eco_LmqsKl 33–12	vB_Sty-LmqsSP6			
*Salmonella* BfR 19-SA02184						
*Salmonella* BfR 20-SA01020						
*Salmonella* BfR 20-SA02231						
*Salmonella* BfR 20-SA00418						
*E. coli* 19/302/1/A						
ESBL *E. coli* 716						
ESBL *E. coli* 290.1						
ESBL *E. coli* 271						
Proportion of Susceptible bacteria	12.50%	25%	12.50%	**62.50%**	**62.50%**	**37.50%**
	**37.50%**					

Plaque formation of the phage cocktail after different stages of the protocol is shown in Table 5. The highest plaque formation was observed after six cycles on *E. coli* 19/302/1/A (8.36 log_10_ PFU/mL) and after 12 cycles on *Salmonella* BfR 20-SA00418 (8.2 log_10_ PFU/mL). The host range of the *Salmonella-*covering phages Lmqs Kl 33–12 and LmqsSP6s changed after six cycles (*Salmonella* BfR 19-SA02184) and after 20 cycles of training (*Salmonella* BfR 20-SA01020 and *Salmonella* BfR 20-SA02231). No plaque formation occurred on any of the ESBLs after starting the training.

**Table 5:** Phage concentrations (log_10_ PFU/mL) of the cocktail determined by spot test after 0, 3, 6, 12, 20, and 30 cycles of the Appelmans protocol on different bacterial strains used for training.

Bacterial strains	0 cycle	3 cycles	6 cycles	12 cycles	20 cycles	30 cycles
*Salmonella* BfR 19-SA02184	0	0	0	6.11	6.23	6.2
*Salmonella* BfR 20-SA01020	0	0	0	5.3	6.08	6.34
*Salmonella* BfR 20-SA02231	0	0	0	3.68	4.74	5.4
*Salmonella* BfR 20-SA00418	7.23	7.34	7.38	8.2	7.43	7.43
*E.coli* 19/302/1/A	7.08	7.67	8.36	7.7	7.95	7.51
ESBL *E. coli* 716	0	0	0	0	0	0
ESBL *E. coli* 290.1	0	0	0	0	0	0
ESBL *E. coli* 271	0	0	0	0	**0**	0
Concentrations determined on the original host bacteria						
*Salmonella* LT2	8.4	7.7	8.29	8.76	8.08	8.52
*E. coli* 3,981/1/22	8.23	0	0	0	0	0
*E. coli* 3617/1/22	8.34	0	0	0	0	0

### 3.3 Antibacterial efficacy of the expanded host range three-phage cocktail in liquid culture

**Figure 1:**
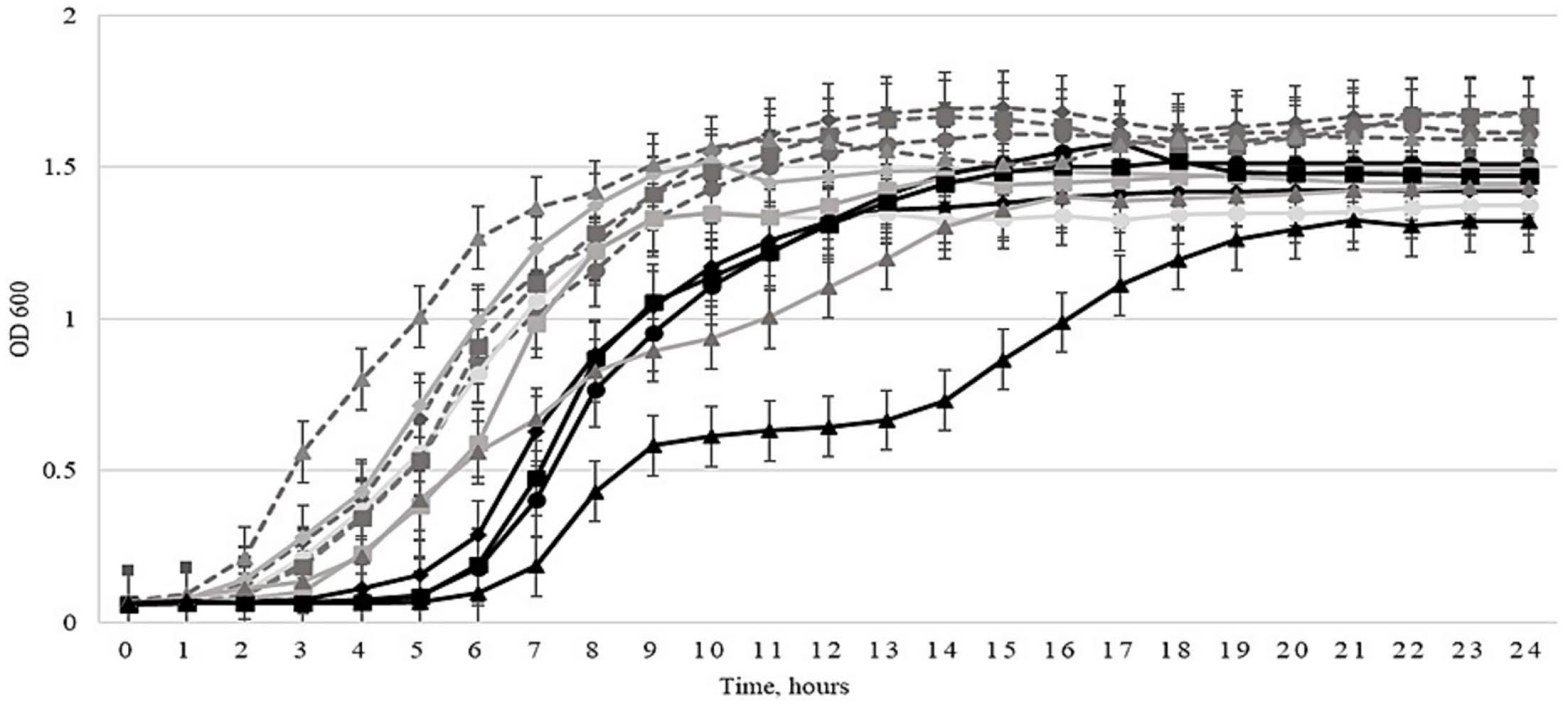
Effects of the three-phage cocktail on liquid cultures of *Salmonella* strains at 0 (light gray solid curves) and after 30 training cycles (black solid curves) using the Appelmans protocol at an MOI of 100. The curves highlighted with different figures: (◊) *Salmonella* BfR 19-SA 2184, (○) *Salmonella* BfR 20-SA1020, (□) *Salmonella* BfR 20-SA02231, (∆) *Salmonella* 20-SA00418. Controls are depicted by light gray dotted curves. Optical density (OD_600_) was measured every hour at 600 nm for 24 h by using the Tecan Spark automatic microplate reader. Error bars represent the standard deviation of three experiments.

**Figure 2:**
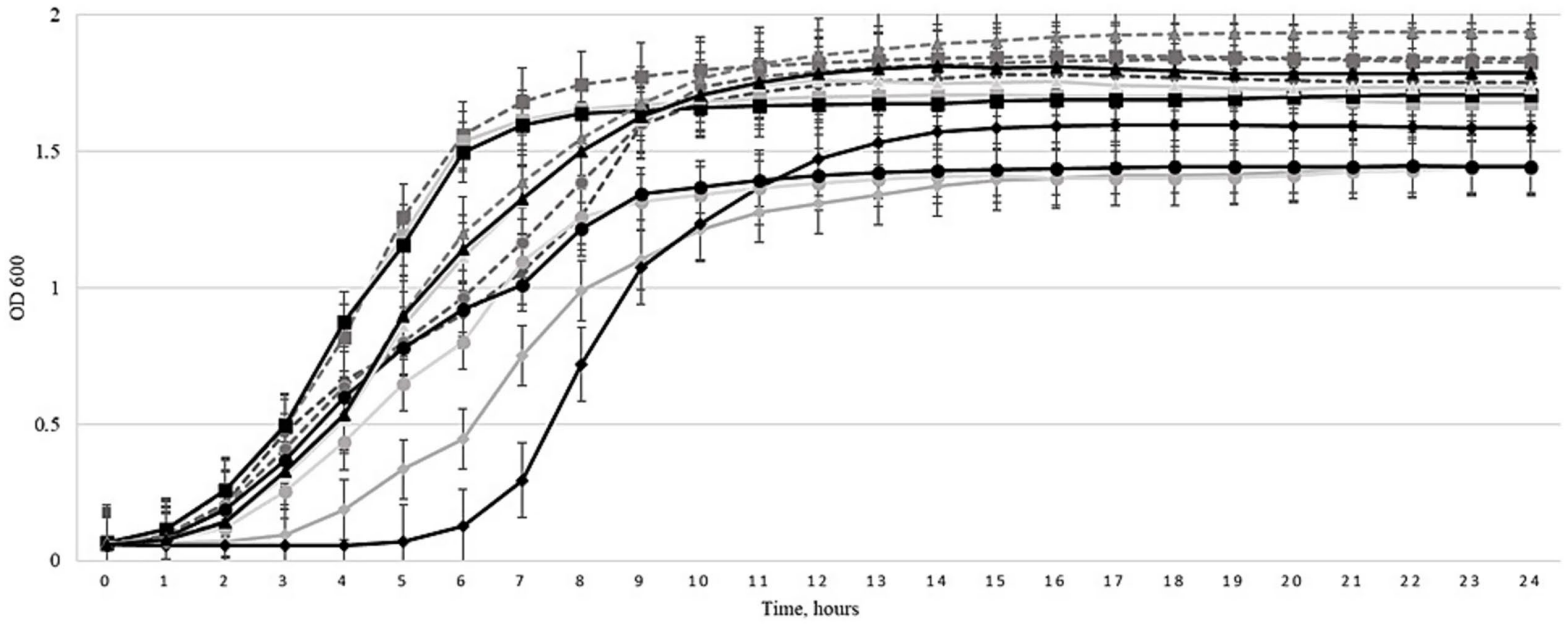
Effects of the three-phage cocktail on liquid cultures of *E. coli* strains at 0 (light gray solid curves) and after 30 training cycles (black solid curves) using the Appelmans protocol at MOI of 100. The curves highlighted with different figures: (◊) *E. coli* 19/302/1/A, (○) ESBL *E. coli* 716, (□) ESBL *E. coli* 290.1, (∆) ESBL *E. coli* 271. Controls are depicted by light gray dotted curves. Optical density (OD_600_) was measured every hour at 600 nm for 24 h by using the Tecan Spark automatic microplate reader. Error bars represent the standard deviation of three experiments.

**Figure 3:**
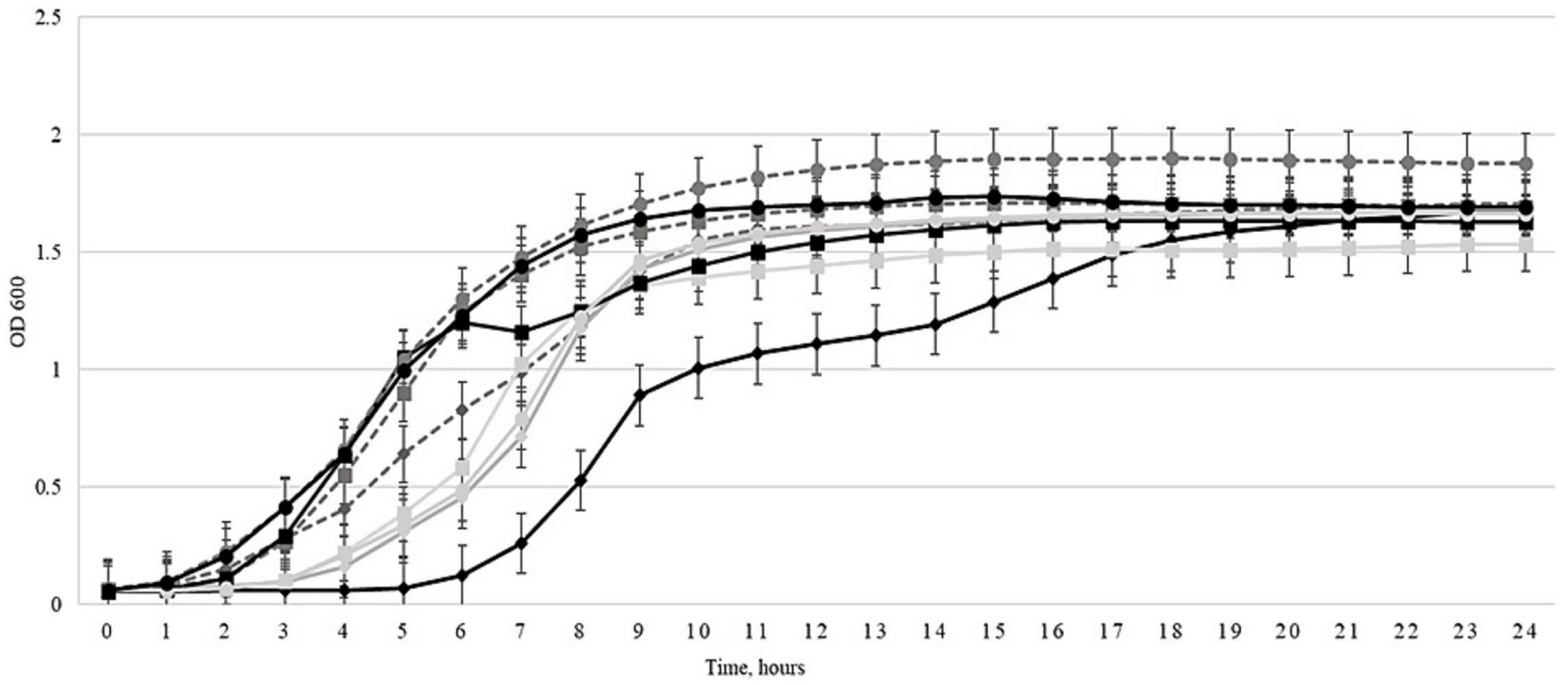
Effects of the three-phage cocktail on liquid cultures of original bacterial host strains at 0 (gray solid curves) and after 30 training cycles (black solid curves) using the Appelmans protocol at an MOI of 100. The curves highlighted with different figures: (◊) *Salmonella* LT2, (□) *E. coli* 3,981 I1 I22, (○) *E. coli* 3,617. Controls are depicted by light gray dotted curves. Optical density (OD_600_) was measured every hour at 600 nm for 24 h by using the Tecan Spark automatic microplate reader. Error bars represent the standard deviation of three experiments.

A planktonic killing assay was conducted to determine the antibacterial efficacy of the expanded three-phage cocktail, comparing its efficacy before and after 30 cycles of Appelmans protocol training. Liquid cultures of 4 *Salmonella* strains and 6 *E. coli* strains were used. Bacterial growth in the presence of the evolved three-phage cocktail compared to growth in the presence of the non-trained control cocktail (before Appelmans training) is shown in Figures 1–3. The evolved three-phage cocktail (30 training cycles) had a significantly longer inhibitory effect on bacterial growth of *Salmonella* strains than the original cocktail before training, as depicted by a shift of the black (trained phage’s) curves to the right. From 2 to 5 h, the reduced growth of *Salmonella* strains was stronger when exposed to the trained cocktail (30 cycles) compared with the original cocktail before training. This was true for all *Salmonella* strains, but the effect weakened at the end of the experiment. However, the inhibitory effect against the *Salmonella* BfR 20-SA 00418 increased from a full growth suppression for 3 h by the initial three-phage cocktail to a growth suppression over a course of 6 h by the trained three-phage cocktail after using Appelmans protocol (Figure 1). However, no improvement in efficacy was observed against most of the tested *E. coli* strains when comparing the trained three-phage cocktail to the cocktail before training, as shown in Figure 2. Even more, none of the cocktails reduced bacterial density compared to the phage-free control (Figure 2). In *E. coli* 19/302/1/A, growth suppression by the trained cocktail was stronger for 8 h compared to the non-trained cocktail, but subsequently, the non-trained cocktail resulted in stronger bacterial inhibition than the trained cocktail for the rest of the experiment (Figure 2). Interestingly, results in Figure 3 show that the growth of *E. coli* host strains was even less efficiently reduced after phage cocktail training compared to the untrained cocktail. However, for the *Salmonella* LT2 strain, the trained three-phage cocktail inhibited growth by approximately 3 h longer than the original cocktail. Regrowth of the *Salmonella* strains was observed after 5 h (Figure 3).

The antimicrobial efficacy of the phage cocktail was evaluated by comparing area under the curve (AUC) values across three conditions: untreated control, cocktail before Appelmans training, and cocktail after 30 cycles of the Appelmans protocol. For *Salmonella* strains, a consistent reduction in AUC values was observed upon treatment with the phage cocktail, with the most significant decreases noted after 30 cycles of Appelmans protocol. For example, the AUC of *Salmonella* BfR 19-SA 2184 declined from 30.5 ± 0.8 in the control group to 27.8 ± 0.9 at 0 cycles and 22.5 ± 1.0 after 30 cycles of the Appelmans protocol. A similar trend was observed in *Salmonella* BfR 20-SA1020, with an AUC of 28.8 ± 1.0, 25.4 ± 0.8, and 22.9 ± 0.9, respectively, and *Salmonella* BfR 20-SA02231 growing at an AUC of 29.3 ± 0.9, 26.0 ± 1.1, and 24.1 ± 0.9. The most notable decrease was seen in *Salmonella* BfR 20-SA00418, where the AUC dropped sharply from 30.9 ± 0.7 in the control group to 23.0 ± 0.9 when grown with the cocktail before training and 16.2 ± 1.1 after 30 cycles of training the three-phage cocktail. For *E. coli* 1930/21/A, AUC values remained relatively stable, with 33.0 ± 1.1 in control, 24.0 ± 1.2 before, and 25.0 ± 1.4 after 30 cycles of training, indicating moderate phage efficacy but no effect by training. The same was true for ESBL *E. coli* 716 (AUC 33.5 ± 0.9 to 26.2 ± 1.0 and 27.5 ± 1.3). Conversely, the *E. coli* strains ESBL 290.1 and ESBL 271 did not show decreased AUC values in the groups with any of the three-phage cocktails compared to the control. Among the original host bacteria of the cocktail, which included phages, *Salmonella* LT2 showed a marked reduction when the trained cocktail was added, with AUC values dropping from 29.7 ± 1.0 in the control and 28.1 ± 0.8 when grown with the non-trained phage cocktail to 22.5 ± 1.1 when grown with the 30-cycle trained phage cocktail. For *E. coli* 3,981 II 122 and *E. coli* 3,617, AUC values were 32.5 ± 0.9, 35.7 ± 0.8, and 26.1 ± 0.7, 29.5 ± 0.9, respectively, for the control and non-trained cocktail groups, and 30.0 ± 1.0 or 31.7 ± 1.2 for the respective strains when the trained cocktail was added.

### 3.4 Effectiveness of the trained three-phage cocktail for controlling *Salmonella* and *E. coli* on raw chicken filets

**Figure 4:**
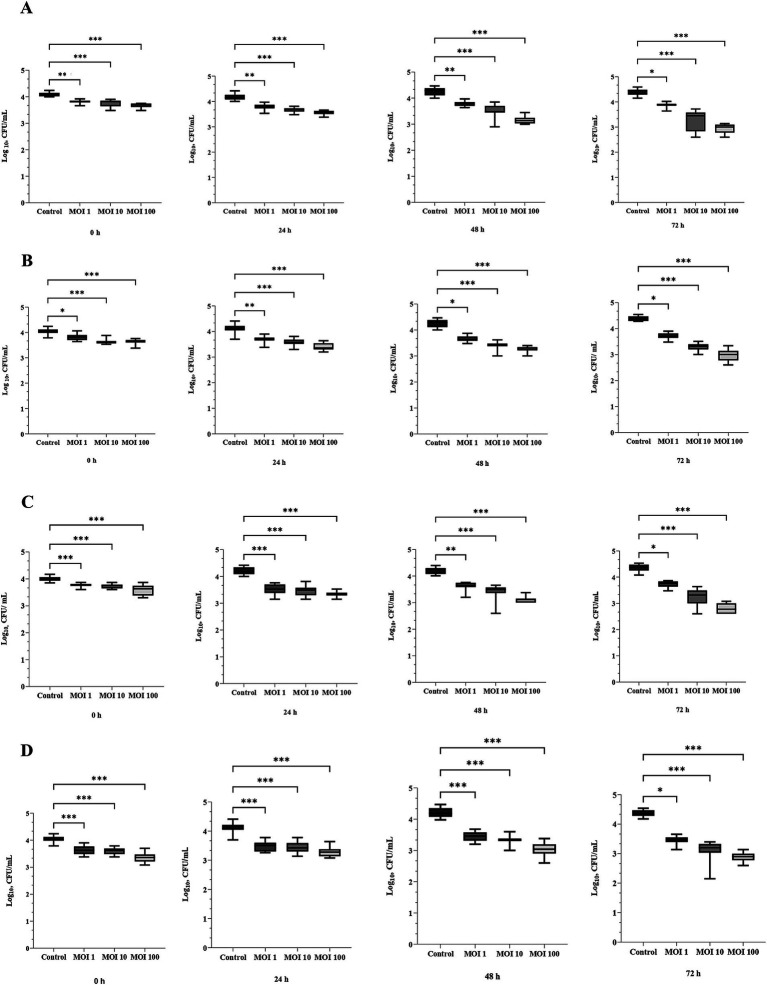
Antibacterial efficacy of the trained three-phage cocktail against **(A)** *Salmonella* BfR 20-SA00418 **(B)** *E. coli* 19/302/1/A, and against a mixture of both bacteria **(C)** *Salmonella* BfR 20-SA00418 and **(D)** *E. coli* 19/302/1/A using different MOIs (*n* = 20). Error bars indicate standard deviation, significance levels are indicated as *p < 0.05; **p < 0.01, ***p < 0.001 compared to the control group.

The efficacy of the trained three-phage cocktail against *Salmonella* and *E. coli* individually and in a mixed contamination model was evaluated on raw chicken filets after 0, 24, 48, and 72 h storage at 4 ± 0.5 °C. In brief, raw chicken filets were cut into pieces and inoculated with *Salmonella* BfR 20-SA00418 (Figure 4A), *E. coli* 19/302/1/A (Figure 4B), or both in combination (Figures 4C,D). Subsequently, experimental groups were treated with the trained three-phage cocktail.

When raw chicken filets were contaminated with *Salmonella* BfR 20-SA00418 only, a slight increase of *Salmonella* load was observed in the control while a dose-dependent decrease was observed after phage treatment. Stable *Salmonella* counts were observed in the MOI 1 phage-treated group and a dose-dependent decrease when higher MOIs were used. After 48 h and later, *Salmonella* counts decreased to 3.5 log_10_ CFU/mL in the MOI 10 and to 3.16 log_10_ CFU/mL in the MOI 100 group, presenting a significant difference compared to the control (*p* < 0.001). The most pronounced *Salmonella* reduction to 2.92 log_10_ CFU/mL compared to the control (*p* < 0.001) was observed with phage treatment at an MOI of 100 after 72 h of storage (Figure 4A).

When *E. coli* 19/302/1/A inoculated raw chicken filets were treated with the trained three-phage cocktail, a dose-dependent decrease was observed in the treatment group. Using MOI 100 resulted in a significant *E. coli* 19/302/1/A decline to 2.99 log_10_ CFU/mL (*p* < 0.001) compared to 4.0 log_10_ CFU/mL in the control at 72 h (Figure 4B).

Bacterial load in raw chicken filets inoculated with a mixture of two target bacteria (*Salmonella* BfR 20-SA00418 and *E.coli* 19/302/1/A) and treated with the trained three-phage cocktail is shown in Figures 4C,D. Phage treatment resulted in a maximum reduction of 1.56 log_10_ CFU/mL *Salmonella* BfR 20-SA00418 (Figure 4C) and 1.48 log_10_ CFU/mL *E.coli* 19/302/1/A (Figure 4D) after 72 h compared to controls when the highest dose (MOI 100) was applied (*p* < 0.001). Overall, as in the other groups, a dose-dependent bacterial reduction was observed when samples were treated with the trained three-phage cocktail (Figures 4A–D).

The measured phage concentrations changed during the challenge experiment on chicken breast filet in a dose-dependent manner. When using the trained three-phage cocktail at MOI 1 in the experimental groups A and B, *Salmonella* BfR 20-SA00418 and *E. coli* 19/302/1/A-specific phages decreased by 0.32 log_10_ PFU/mL and 0.49 log_10_ PFU/mL, respectively, after 72 h compared to the initial level. When an MOI 10 was used, different outcomes in phage titers were observed in the experimental groups A (*Salmonella* BfR 20-SA00418) and B (*E. coli* 19/302/1/A). After 72 h of cold storage of raw chicken filets, the phage titers changed from 5 log_10_ PFU/mL to 4.94 log_10_ PFU/mL in the *Salmonella*-only challenge and to 5.2 log_10_ PFU/mL in the *E. coli*-only experiment. When MOI 100 was used, phage titers in these experimental groups decreased by 0.12 log_10_ PFU/mL and 0.8 log_10_ PFU/mL, respectively. When phages were applied on raw chicken filets contaminated with a mixture of two tested strains (experimental groups C and D), a slight increase after 72 h was observed, 0.1 log_10_ PFU/mL for MOI 1 and 0.14 log_10_ PFU/mL for MOI 100 treated groups were measured on *Salmonella* BfR 20-SA00418 and 0.07 log_10_ PFU/mL and 0.32 log_10_ PFU/mL at MOI 1 and 10 on *E.coli* 19/302/1/A.

## 4 Discussion

Phage-based biocontrol is increasingly considered as a future strategy for reducing food-contaminating bacteria. However, one main limitation for the widespread application of phages in food biocontrol is the narrow host range of a single phage against bacterial strains. Isolating phages from natural sources, it is hard to obtain single phages that effectively lyse multiple bacterial strains or even species. The combination of a single phage with a wide spectrum of host ranges in the cocktails is under discussion to solve this problem (de Melo et al., 2018; Połaska and Sokołowska, 2019; Ahmadi et al., 2020). Several studies have reported the successful application of phage cocktails against *E. coli* and *Salmonella* (Shahdadi et al., 2023; Gvaladze et al., 2024; Kim and Lim, 2025) and other foodborne pathogens on various meat types, including poultry. Some phage cocktails for (Boyacioglu et al., 2016; Carter et al., 2012) *E. coli* or *Salmonella* (Woolston et al., 2013) control in food are being commercially used. However, limited studies are available on polyvalent phage cocktail application against multi-species *Salmonella* and *E. coli* contamination on poultry meat (Poojari et al., 2022). The main aim of the present study was to develop an effective phage cocktail for simultaneously controlling *Salmonella* and *E. coli* on raw chicken filets during cold storage.

The approach of combining phages in cocktail formulations has been widely used in phage therapy and biocontrol research (Islam et al., 2019; Yang et al., 2020; Torkashvand et al., 2024; Yoo et al., 2024). Furthermore, it is worth mentioning that previous studies suggest that a mixture of phages during phage cocktail design helps not only broaden the spectrum of activity but also help mitigate resistance development in bacterial populations (Chan et al., 2013; Khan Mirzaei and Nilsson, 2015; Martinez-Soto et al., 2024). Furthermore, as noted by Abedon et al. (2021), phages with modest EOP values can still enhance bacterial suppression in cocktails through additive or synergistic effects, especially when targeting subpopulations with different resistance profiles. Based on these hypotheses, our selection strategy for combining phages focused on those with varying host ranges, target specificity, and EOP grades, thereby enhancing the overall efficacy of the phage cocktail. During this study, three phages were selected based on the results of host range and EOP analysis to be included in an Appelmans protocol phage cocktail training. The selected phages exhibited the following characteristics: phage vB_Eco_LmqsKl31-21, targeting 60% of *E. coli,* including ESBL strains, phage vB_Eco_LmqsKl33-12, which displayed activity against both *E. coli* (30.0%) and *Salmonella* (7.7%) strains with moderate and high EOP values, respectively, and phage vB_Sty-LmqsSP6, which had specific moderate sensitivity to a *Salmonella* strain (7.7%). Four *Salmonella* and four *E. coli* (including three ESBL) were selected, including propagating and minor-susceptible or non-susceptible strains for all phages. Their characteristics are shown in Table 3.

Phage training has been proposed as a revisited tool that uses the evolutionary potential of phages, improving their infectivity, expanding host range, overcoming bacterial resistance, and enhancing their stability under different environmental conditions, based on the training settings used. Various methods for phage training have been developed under laboratory conditions (Borin et al., 2021; Borges, 2021; Bull et al., 2022). However, the Appelmans protocol is gaining attention for its potential to expand the host range of single phages and their mixtures (cocktails), to evolve and adapt bacteriophages for infecting previously resistant or non-susceptible bacterial strains (Burrowes et al., 2019; Vu et al., 2024). We used 30 cycles of the Appelmans protocol to extend the host range of our three-phage cocktail, and our results show an increased host range after training, infecting 5/8 (62.50%) instead of 3/8 (37.50%) of the bacterial strains tested (Table 4). Vu et al. (2024) reported an expanded host range for a phage cocktail targeting CRAB after 40 cycles, achieving lysis in 80% of the strains used. Furthermore, Burrowes et al. (2019) reported a maximum expanded host range of the phage cocktail to the 10 bacterial strains (100%) compared to two susceptible bacterial strains before the experiment, after 30 cycles of the Appelmans protocol with three input phages against *P. aeruginosa*.

The antibacterial efficacy of the trained three-phage cocktail was assessed using a planktonic killing assay. The results presented in Figures 1–3 demonstrate that the three-phage cocktail exhibited significantly enhanced killing activity after 30 training cycles compared to the untrained cocktail (0 cycles). The inhibitory effect on bacterial growth was pronounced during the initial 3–6 h in *Salmonella* and *E. coli* tested strains. In particular, the trained three-phage cocktail reduced all tested *Salmonella* strains, lasting for at least 3 h (4/4, 100%). *Salmonella* BfR 20-SA00418 and *Salmonella* LT2 growth suppression lasted longer, and regrowth occurred only after 6 h. Conversely, *E.coli* strains treated with the three-phage cocktail, showed no growth inhibition compared to the non-trained cocktail treated group. However, some showed reduced growth compared to the control and, *E. coli* 19/302/1/A showed no growth for 5 h. The regrowth of tested bacteria after 5–6 h when grown with the trained three-phage cocktail can be explained by infection efficacy and concentration of the phages. We used a MOI of 1, obviously leading to a remaining proportion of non-infected bacteria that were able to replicate and sustain regrow after few hours incubation. Our results contrast with those of a previously published study, which reported the efficacy of an untreated phage cocktail against *S. enterica* using a similar assay and completely suppressed bacterial growth within the first 2 h using MOIs of 0.01, 1, 100, 1,000, and 10,000. However, bacterial levels began to increase again after 4 h, potentially owing to the emergence of resistance (Torkashvand et al., 2024).

Several studies have reported effectiveness of phages and phage cocktails for *Salmonella* and *E. coli* mitigation in experimental meat models using phage treatment (Kumar et al., 2017; Poojari et al., 2022; Gvaladze et al., 2024; Almutairi et al., 2022). This is the first study to examine phage cocktails for controlling these two important foodborne pathogens simultaneously on a meat model. Using a phage combination has the potential to increase the chance of successful bacterial eradication. Each phage targets different bacterial receptors or exhibits different mechanisms of action, thereby reducing the likelihood of bacterial resistance development (Jassim and Limoges, 2014). Moreover, studies have demonstrated that phage cocktails targeting *E. coli* and *Salmonella* can achieve higher log reductions compared to single-phage treatments (Costa et al., 2019). According to the literature, temperature and timing of phage application as well as phage and bacterial concentrations (as stated giving the MOI) play a significant role in the success of phage treatment. Many papers describe the use of different concentrations, volumes, and doses as well as the timings of phage application (Kim et al., 2020; Huang et al., 2022). In most papers, the MOI levels ranged from 0.001 and 1 to 10^2^–10^8^ (Minh et al., 2016; Seo et al., 2016; Sukumaran et al., 2015; Kim et al., 2020). Furthermore, phage titers dropped during our experiments. However, this can be explained by lower phage replication compared to the loss of free phages by inactivation and infection. It could mean that during long-term storage, only at the beginning, effective phages were present in sufficient concentration. However, as in other predator–prey relationships, population density fluctuates in phases, and reduced phage concentrations may only represent a snapshot in time.

In the present investigation, raw chicken filets were used as representative meat models frequently contaminated with *Salmonella* and *E. coli* to evaluate the efficacy of a phage cocktail controlling these pathogens. Our results indicate that the developed and optimized three-phage cocktail was effective in reducing the viable counts of tested bacteria in meat samples and showed the strongest lytic activity against *Salmonella* BfR 20-SA00418 and less pronounced against *E.coli*19/302/1/A. The trained three-phage cocktail significantly reduced the viable counts of tested bacteria at MOI 100 at 4 ± 0.5 °C after 72 h when compared to controls. However, despite the broadened host range of the trained cocktail, some efficacy against *E. coli* was lost, while efficacy against *Salmonella* was increased. Future studies should focus on developing methods that reduce such payoffs during phage training.

## 5 Conclusion

In summary, we selected three lytic phages, developed a three-phage cocktail targeting *Salmonella* and *E. coli* and optimized it by Appelmans protocol. The trained three-phage cocktail demonstrated improved efficacy to control *Salmonella* and some *E. coli* on raw chicken filets at storage conditions. Overall, our findings suggest that the developed and optimized three-phage cocktail can be used for the biocontrol of mixed *Salmonella* and *E.coli* contamination in chicken meat, enhancing food safety in chicken meat production.

## Data Availability

The original contributions presented in the study are included in the article/supplementary material; further inquiries can be directed to the corresponding author.
