# Supplementary material for: Phage-based biocontrol of Salmonella and E. coli in raw chicken filets: optimizing phage-based solutions to enhance food safety under cooled storing conditions
Source: Front Microbiol. 2026 Jan 7;16:1696826. doi: 10.3389/fmicb.2025.1696826 (PMC12819821; doi:10.3389/fmicb.2025.1696826)
Supplement: Supplementary file 1 [file Table_1.docx]

Supplementary Material

**Supplementary Table S1.** Bacterial strains used for the study with a brief summary of their characteristics (n=23).

|  | **Isolate ID** | **Serotype and characteristics** |
| --- | --- | --- |
| 1 | **BfR 19-SA02184** | ***S.* Indiana** |
| 2 | **BfR 20-SA02878** | ***S.* Typhimurium** |
| 3 | **BfR 20-SA02511** | ***S.* Infantis** |
| 4 | **BfR 20-SA01020** | ***S.* Typhimurium** |
| 5 | **BfR 19-SA01081** | ***S.* Paratyphi B** |
| 6 | **BfR 19-SA00115** | ***S.* Enteritidis** |
| 7 | **BfR 20-SA02231** | ***S.* Enteritidis** |
| 8 | **BfR 20-SA02265** | ***S.* Infantis.** |
| 9 | **BfR 20-SA01326** | ***S.* Paratyphi B** |
| 10 | **BfR 20-SA01985** | ***S.* Indiana.** |
| 11 | **BfR 20-SA00418** | **S*. subsp. enterica rough variant*** |
| 12 | **Salmonella 11** | ***S. Serogroup C*** |
| 13 | **Salmonella LT2** | ***S.* Typhimurium** |
| 14 | **19/302/1/A** | ***E. coli* O2 (serotype).** Sinus infraorbitalis. Turkey.  Host bacterium of phage vB_Eco_LmqsGe **33-1**. |
| 15 | **2763/1/22** | ***E. coli*.** Host bacterium of phage vB_Eco_LmqsKl **33-33**. |
| 16 | **3981/1/22** | ***E. coli*.** Host bacterium of phage vB_Eco_LmqsKl **33-19.** |
| 17 | ***E. coli* 28** | ***E. coli.*** Host bacterium of phage **G 28** |
| 18 | **ESBL** *E.coli* **715** | ***E. coli*.** Processed chicken meat. Shows resistance for Cefotaxim. |
| 19 | **ESBL** *E.coli* **716** | ***E. coli*.** Processed chicken meat. Shows resistance for Cefotaxim. |
| 21 | **ESBL** *E.coli* **290.1** | ***E. coli*.** Chicken breast fillet. Shows resistance for Ceftazidim and Cefotaxim. |
| 22 | **ESBL** *E.coli* **290.2** | ***E. coli*.** Chicken breast fillet. Shows resistance for Ceftazidim and Cefotaxim. |
| 23 | **ESBL** *E.coli* **365** | ***E. coli*.** Chicken breast fillet. Shows resistance for Cefepim, Ceftazidim and Cefotaxim. |

**Supplementary Table S2.** Phages used for the study with a brief summary of their characteristics (n=17).

|  | **Phage ID** | **Strain for propagation** | **Origin** |
| --- | --- | --- | --- |
| **1** | **vB_Eco_LmqsRi 2-3** | *E. coli* A1349/20 | Manure |
| **2** | **vB_Eco_LmqsRi 2-6** | *E. coli* A602/21 | Manure |
| **3** | **vB_Eco_LmqsRi 2-8** | *E. coli* A1500/21 | Manure |
| **4** | **vB_Eco_LmqsRi 4-3** | *E. coli* A1349/20 | Manure |
| **5** | **vB_Eco_LmqsRi 6-1** | *E. coli* A1349/20 | Manure |
| **6** | **vB_Eco_LmqsGe 33-1** | *E. coli* 19\|302\|1\|A | Waste water |
| **7** | **vB_Eco_LmqsKl 31-18** | *E. coli* 3913 I4 I22 | Waste water |
| **8** | **vB_Eco_LmqsKl 31-21** | *E. coli* 3981 I1 I22 | Waste water |
| **9** | **vB_Eco_LmqsKl 31-27** | *E. coli* 6093 I1 I22 | Waste water |
| **10** | **vB_Eco_LmqsKl 33-6** | *E. coli* 1363 I1 I22 | Waste water |
| **11** | **vB_Eco_LmqsKl 33-12** | *E. coli* 3617 I1I22 | Waste water |
| **12** | **vB_Eco_LmqsKl 33-19** | *E. coli* 3981 I1 I22 | Waste water |
| **13** | **vB_Eco_LmqsKl 33-21** | *E. coli* 5358 I1 I22 | Waste water |
| **14** | **vB_Eco_LmqsKl 33-27** | *E. coli* S5143/2 | Waste water |
| **15** | **vB_Eco_LmqsKl 33-33** | *E. coli* 2763/1/22 | Waste water |
| **16** | **Phage G 28** | *E. coli* 28 | Manure |
| **17** | **vB_Sty-LmqsSP6** | *Salmonella* LT2 | Nasal swab of cow |

**Supplementary Table S3.** Results of host range determination

| Bacterial isolates |  |  |  |  |  |  |  |  |  |  |  |  |  |  |  |  |
| --- | --- | --- | --- | --- | --- | --- | --- | --- | --- | --- | --- | --- | --- | --- | --- | --- |
|  | vB_Eco_LmqsRi 2-3 | vB_Eco_LmqsRi 2-6 | vB_Eco_LmqsRi 2-8 | vB_Eco_LmqsRi 4-3 | vB_Eco_LmqsRi 6-1 | vB_Eco_LmqsGe 33-1 | vB_Eco_LmqsKl 31-18 | vB_Eco_LmqsKl 31-21 | vB_Eco_LmqsKl 31-27 | vB_Eco_LmqsKl 33-6 | vB_Eco_LmqsKl 33-12 | vB_Eco_LmqsKl 33-19 | vB_Eco_LmqsKl 33-21 | vB_Eco_LmqsKl 33-27 | vB_Eco_LmqsKl 33-33 | vB_Sty-LmqsSP6 |
| *Salmonella* BfR 19-SA02184 |  |  |  |  |  |  |  |  |  |  |  |  |  |  |  |  |
| *Salmonella* BfR 20-SA02878 |  |  |  |  |  |  |  |  |  |  |  |  |  |  |  |  |
| *Salmonella* BfR 20-SA02511 |  |  |  |  |  |  |  |  |  |  |  |  |  |  |  |  |
| *Salmonella* BfR 20-SA01020 |  |  |  |  |  |  |  |  |  |  |  |  |  |  |  |  |
| *Salmonella* BfR 19-SA01081 |  |  |  |  |  |  |  |  |  |  |  |  |  |  |  |  |
| *Salmonella* BfR 19-SA00115 |  |  |  |  |  |  |  |  |  |  |  |  |  |  |  |  |
| *Salmonella* BfR 20-SA02231 |  |  |  |  |  |  |  |  |  |  |  |  |  |  |  |  |
| *Salmonella* BfR 20-SA02265 |  |  |  |  |  |  |  |  |  |  |  |  |  |  |  |  |
| *Salmonella* BfR 20-SA01326 |  |  |  |  |  |  |  |  |  |  |  |  |  |  |  |  |
| *Salmonella* BfR 20-SA01985 |  |  |  |  |  |  |  |  |  |  |  |  |  |  |  |  |
| *Salmonella* BfR 20-SA00418 |  |  |  |  |  |  |  |  |  |  |  |  |  |  |  |  |
| *Salmonella* 11 |  |  |  |  |  |  |  |  |  |  |  |  |  |  |  |  |
| *Salmonella* LT2 |  |  |  |  |  |  |  |  |  |  |  |  |  |  |  |  |
| *E. coli* 19/302/1/A |  |  |  |  |  |  |  |  |  |  |  |  |  |  |  |  |
| *E. coli* 2763/1/22 |  |  |  |  |  |  |  |  |  |  |  |  |  |  |  |  |
| *E. coli* 3981/1/22 |  |  |  |  |  |  |  |  |  |  |  |  |  |  |  |  |
| *E. coli* 28 |  |  |  |  |  |  |  |  |  |  |  |  |  |  |  |  |
| *E. coli* ESBL 715 |  |  |  |  |  |  |  |  |  |  |  |  |  |  |  |  |
| *E. coli* ESBL 716 |  |  |  |  |  |  |  |  |  |  |  |  |  |  |  |  |
| *E. coli* ESBL 290.1 |  |  |  |  |  |  |  |  |  |  |  |  |  |  |  |  |
| *E. coli* ESBL 290.2 |  |  |  |  |  |  |  |  |  |  |  |  |  |  |  |  |
| *E. coli* ESBL 365 |  |  |  |  |  |  |  |  |  |  |  |  |  |  |  |  |
| *E. coli* ESBL 271 |  |  |  |  |  |  |  |  |  |  |  |  |  |  |  |  |
| Sensitive  strains/23 tested strains, (%) | 0 | 0 | 1  (4) | 0 | 0 | 2  (9) | 2  (9) | 6  (26) | 3  (13) | 2  (9) | 4  (17) | 6  (26) | 3  (13) | 4  (17) | 6  (26) | 1  (4) |

Phage/bacterial combinations that produced visible plaques in three replicates were used to calculate the efficiency of plat󠄗ing (EOP). Color coding was used to visualize the EOP: 󠄀 red: high sensitivity (EOP ≥ 0.5); light orange: moderate sensitivity (0.1 ≤ EOP ≤ 0.5); 󠄀light pink: low sensitivity (0.001≤ EOP  ≤ 0.1). White fields indicate no lysis. A heat map was generated using a table prepared in Microsoft Word
